# Correlating Molecular Precursor Interactions with Device Performance in Solution-Processed Cu_2_ZnSn(S,Se)_4_ Thin-Film Solar Cells

**DOI:** 10.1021/acsami.4c05321

**Published:** 2024-06-27

**Authors:** Raphael Agbenyeke, Alice Sheppard, Jacques Keynon, Nada Benhaddou, Nicole Fleck, Valentina Corsetti, Mohammed A. Alkhalifah, Devendra Tiwari, Jake W. Bowers, David J. Fermin

**Affiliations:** † School of Chemistry, University of Bristol, Cantocks Close, Bristol BS8 1TS, U.K.; ‡ Centre for Renewable Energy Systems Technology (CREST), Wolfson School of Mechanical, Electrical and Manufacturing Engineering, Loughborough LE11 3TU, U.K.; § Department of Mathematics, Physics and Electrical Engineering, Northumbria University, Ellison Building, Newcastle Upon Tyne NE1 8ST, U.K.; ∥ Department of Chemistry, College of Science, King Faisal University, Al-Ahsa 31982, Saudi Arabia

**Keywords:** Cu_2_ZnSn(S,Se)_4_, solution processing, thin-film PV, cation complexation, thiourea, colloids

## Abstract

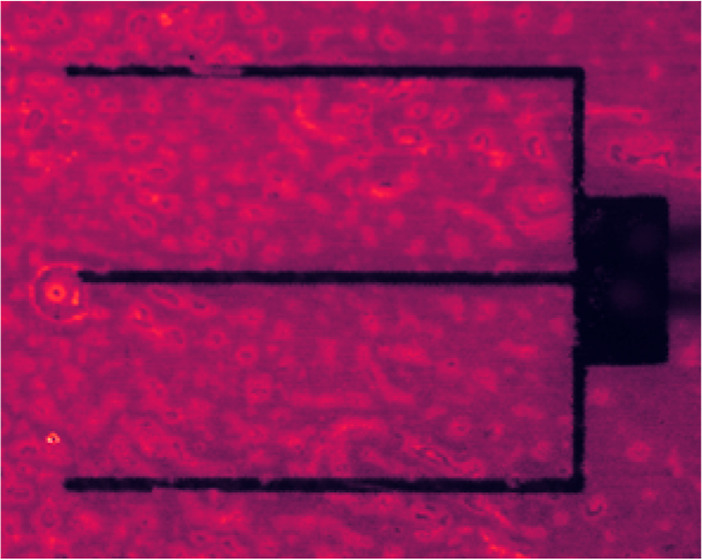

Research efforts aimed at improving the crystal quality of solution-processed Cu_2_ZnSn(S,Se)_4_ (CZTSSe) absorbers have largely employed delicate pre- and postprocessing strategies, such as multistep selenization, heat treatment in mixed chalcogen atmospheres, and multinary extrinsic doping that are often complex and difficult to reproduce. On the other hand, understanding and tuning chemical interactions in precursor inks prior to the thin-film deposition have received significantly less attention. Herein, we show for the first time how the complexation of metallic and chalcogen precursors in solution have a stark influence on the crystallization and optoelectronic quality of CZTSSe absorbers. By varying thiourea to metal cation ratios (TU/M) in dimethylformamide (DMF) and isopropyl alcohol (IPA)-based inks, we observed the formation of nanoscale metal–organic complexes and submicron size aggregates which play a key role in the morphology of the precursor layers obtained by spin-coating and drying steps. We also identify the primary cations in the complexation and assembling processes in solution. The morphology of the precursor film, in turn, has an important effect on grain growth and film absorber structure after the reactive annealing in the presence of Se. Finally, we establish a link between metal complexes in precursor solutions and device performance, with power conversion efficiency increasing from approximately 2 to 8% depending on the TU/M and Cu/(Zn + Sn) ratios.

## 1 Introduction

The rapidly growing demand for electricity and the urgency to transition to green energy solutions have intensified research efforts in renewable energy technologies. According to the world energy outlook, renewable energy alternatives are expected to contribute up to 80% of new power generation by 2030, with photovoltaics accounting for more than half of this growth.^1^ Silicon-based modules occupy the largest portion of the current PV market and will therefore play a crucial role in reaching the goal of a carbon neutral economy. However, the rapid approach of the Si technology to its fundamental power conversion efficiency (PCE) limit of 29.1%,^2^ as well as inherent material rigidity and heavy weight, limit advanced applications, such as building and vehicle-integrated photovoltaics.^3,4^ This has motivated interest in thin-film photovoltaic technologies with high flexibility and potential for low-cost manufacturing. Cu_2_ZnSn(S,Se)_4_ (CZTSSe), is one of these technologies leveraging attractive material properties, such as composition tunable band gap, high absorption coefficient, and intrinsic p-type conductivity, as well as low material cost and solution processability suitable for large-scale deployment. The lab-scale PCE of CZTSSe solar cells is steadily approaching the threshold for commercial viability after a long period of stagnancy around 12.6%.^5^ This encouraging trend has been driven by a concentrated focus on minimizing Voc deficit using different strategies including extrinsic doping,^6−9^ multistep heat treatment,^10,11^ and surface conditioning or interfacial passivation.^12,13^

Solution-based deposition of precursor films is not only an attractive strategy toward scalable processing of CZTSSe films but also a versatile approach to incorporating dopants and additives.^14^ A representative example is the use of hydrazine by Wang et al., which led to a record device for many years.^5^ In this approach, solutions containing metal cations and sulfur precursors are deposited by methods such as spin-coating, inject printing, slot die, and spray coating onto the substrate generating a dry precursor film.^14^ These films are subsequently annealed in the presence of Se to generate polycrystalline CZTSSe films. By far, studies on precursor optimization have primarily focused on solvent mixtures. Ki et al. introduced dimethyl sulfoxide (DMSO)-based solvent systems, which is now commonly used to produce high efficiency cells.^15−18^ Meng et al. have also obtained high PCE devices using 2-methoxyethanol (2-MeO) solvent systems.^19−21^ Guo et al. reported a detailed investigation of metal oxide precursors complexation with thioglycolic acid (TGA) in aqueous base solution, generating devices with PCE comparable to those obtained from hydrazine-based precursor.^22^ More recently, Xu et al. showed the potential of reaching PCE above 13% across a wide elemental composition window using TGA, suggesting a preferential interaction between Sn ions and TGA to form large metal–organic molecules, which decompose during selenization into a low resistance, high workfunction graphitic interlayer ideal for charge transport.^23^

In this report, we demonstrate for the first time the complex link between the chemistry associated with cation complexation and self-assembly in precursor inks with a thin-film morphology and device performance. The key innovative aspect of our study is the connection between thiourea (TU) metal complexes, their self-assembly into larger aggregates, and how these affect the microstructure of the films prior (dry precursor) and after reactive annealing in the presence of Se. FTIR and Raman spectroscopy show clear evidence of selective metal complexation by TU used as a sulfur precursor in solution. By varying the concentration ratio TU to metal cations (TU/M), we observe a systematic increase in the size of metal complexes in solution, which translates into a range of morphologies at the dry precursor level and after reactive annealing/selenization. We demonstrate a correlation between cation complexation, precursor morphology, grain growth and device performance, with the best cell exhibiting a short-circuit current (*J*_SC_), open-circuit voltage (*V*_OC_), fill factor (FF), and PCE of 31.4 mA cm^–2^, 0.403 V, 0.63, and 7.95%, respectively. Our data conclusively show that ink formulation for solution-based deposition of semiconductors plays a role much more important than just delivering the elements required for crystal growth.

## 2 Results and Discussion

### 2.1 From Colloidal Structures to Thin Films

**Figure 1:**
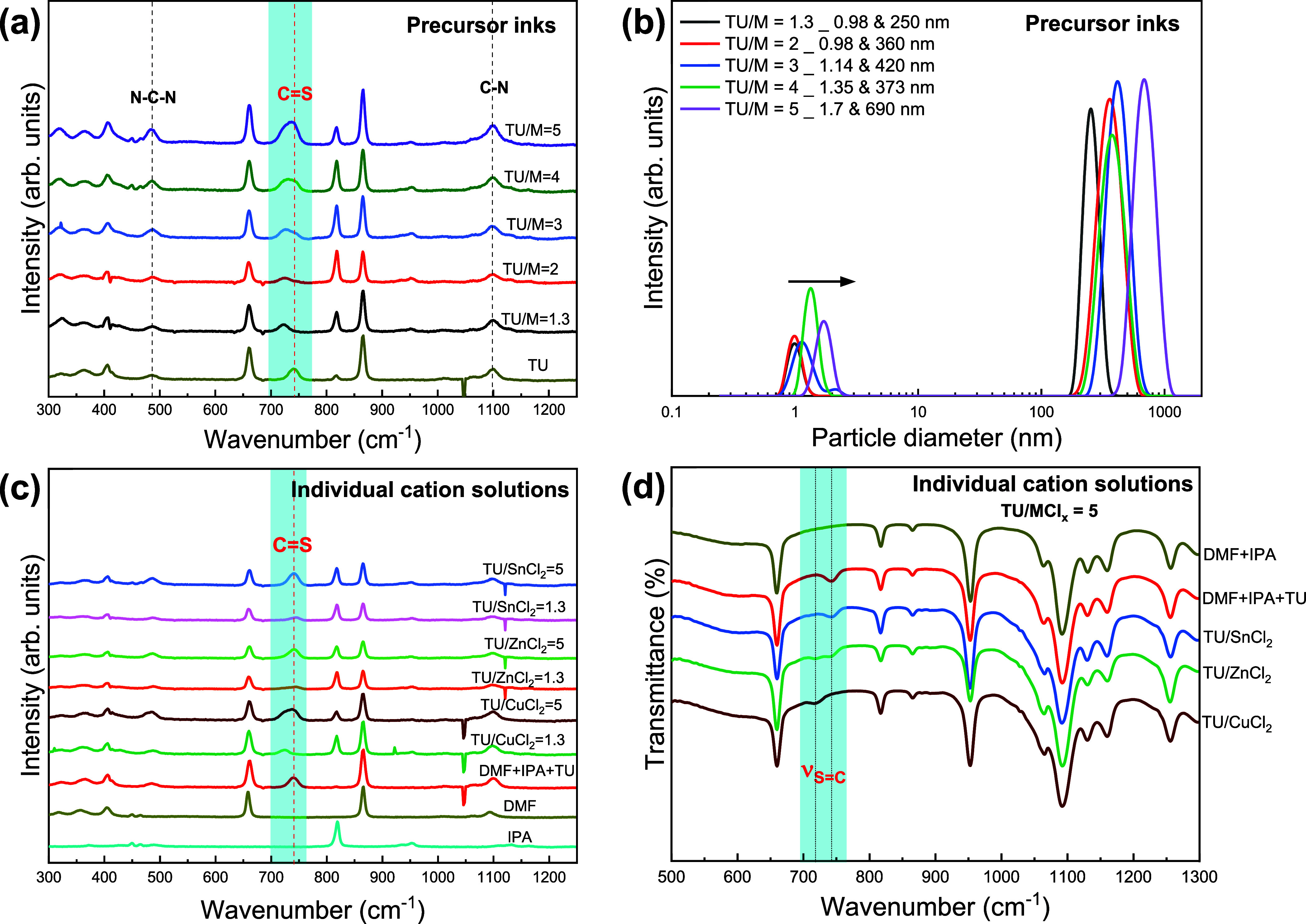
Metallic complexation in precursor inks: (a) Raman spectra of different ratios of thiourea (TU) and metal ions dissolved in a DMF/IPA solvent mixture. (b) Hydrodynamic particle diameter distribution of the precursor ink as a function of TU/M ration as probed by dynamic light scattering measurements. (c) Raman spectra of individual metal salts and TU dissolved in DMF/IPA. (d) FTIR spectra of metal salts and TU dissolved in DMF/IPA.

Figure 1a depicts the Raman spectra of the precursor complexes in a 1:1 mixture of dimethylformamide (DMF)/isopropyl alcohol (IPA) as solvent with different thiourea-to-metal ratios (TU/M), as well as a reference solution containing only thiourea (TU). The peaks at 486, 741, and 1085 cm^–1^ corresponding to the N–C–N bending, C=S stretching, and C–N stretching Raman modes confirmed the presence of TU in the reference solution, while all other peaks were indexed to DMF and IPA. In the CZTS precursor inks, the C=S peak red-shifted to 723 cm^–1^, indicating an extension in bond length owing to chemical coordination with the metal ions in solution.^24,25^ The peak broadened toward higher TU concentrations, followed by the emergence of the shoulder peak around 740 cm^–1^, which steadily grew in intensity. No shifts were observed in the N–C–N and C–N peaks, suggesting negligible chemical interactions between these groups and the metal ions.

Dynamic light scattering measurements on the same inks revealed two colloidal size domains in the nano and submicrometer scale, as shown in Figure 1b. The colloids with hydrodynamic diameters in the range of 0.98 to 1.7 nm are attributed to molecular complexes, which self-assemble into colloidal particles with relatively narrow size distributions. Figure 1b clearly shows that both colloidal size domains increase with increasing TU concentration, suggesting multiple coordination between metal ions and TU. Indeed, previous studies have shown that solvated CuCl_2_ can interact with up to three TU anions to form stable complexes.^26^ Another remarkable observation is the shift of the maximum of submicrometer colloidal assembly from 250 to 690 nm as the TU/M ratio increases from 1.3 to 5. This result suggests an equilibrium among molecular complexes, colloidal assemblies, and free TU in the molecular precursor. Although we do not have detailed information about the structures of these assemblies, we will show that these have a clear impact on the microstructure of the CZTSSe films. It should also be mentioned that the stability of the colloidal solution increases from a few hours to weeks upon increasing the TU/M from 1.3 to 5, with no color changes or precipitation.

To elucidate the role of each metal salt in the complexation process, separate solutions of CuCl_2_, ZnCl_2_, and SnCl_2_ with TU dissolved in DMF/IPA mixtures were probed by Raman spectroscopy, as shown in Figure 1c. The C=S signal in the CuCl_2_ solution red-shifted from the reference position at low TU concentration and exhibited a double peak at a higher concentration, confirming CuCl_2_ complexation by TU. By contrast, symmetric peaks with no shifts were observed in the presence of ZnCl_2_ and SnCl_2_ solutions, suggesting no chemical interaction with TU. Figure 1d shows the FTIR spectra of the metal cations in the presence of TU. The C=S absorption peak at 742 cm^–1^ is shifted to 718 cm^–1^ in the CuCl_2_ solution, while no shifts were observed for the SnCl_2_ solution, which is in good agreement with the Raman spectra. Interestingly, two peaks associated with TU were observed for the ZnCl_2_ solution, one at 742 cm^–1^ corresponding to uncoordinated TU and the other at 718 cm^–1^ representing TU species chemically coordinated with ZnCl_2_. Indeed, it has also been reported that Zn(II) cation can coordinate up to four TU anions in solution.^27^ This indicates the participation of Zn ions in the complexation process and suggests a higher sensitivity of FTIR over Raman spectroscopy in identifying these chemical species. As depicted in Figure S1, the peak at 718 cm^–1^ was not detected at low concentrations of TU (TU/M = 1.3), further confirming a stronger chemical interaction between Cu and S as suggested in previous reports.^28^ Raman and IR data allow concluding a selective formation of CuCl_2_-TU complexes at low TU concentrations, while ZnCl_2_-TU species are formed at higher concentrations, which is responsible for the formation and self-assembly of colloidal species (cf. Figure 1a) and the stability of the precursor solution.

**Figure 2:**
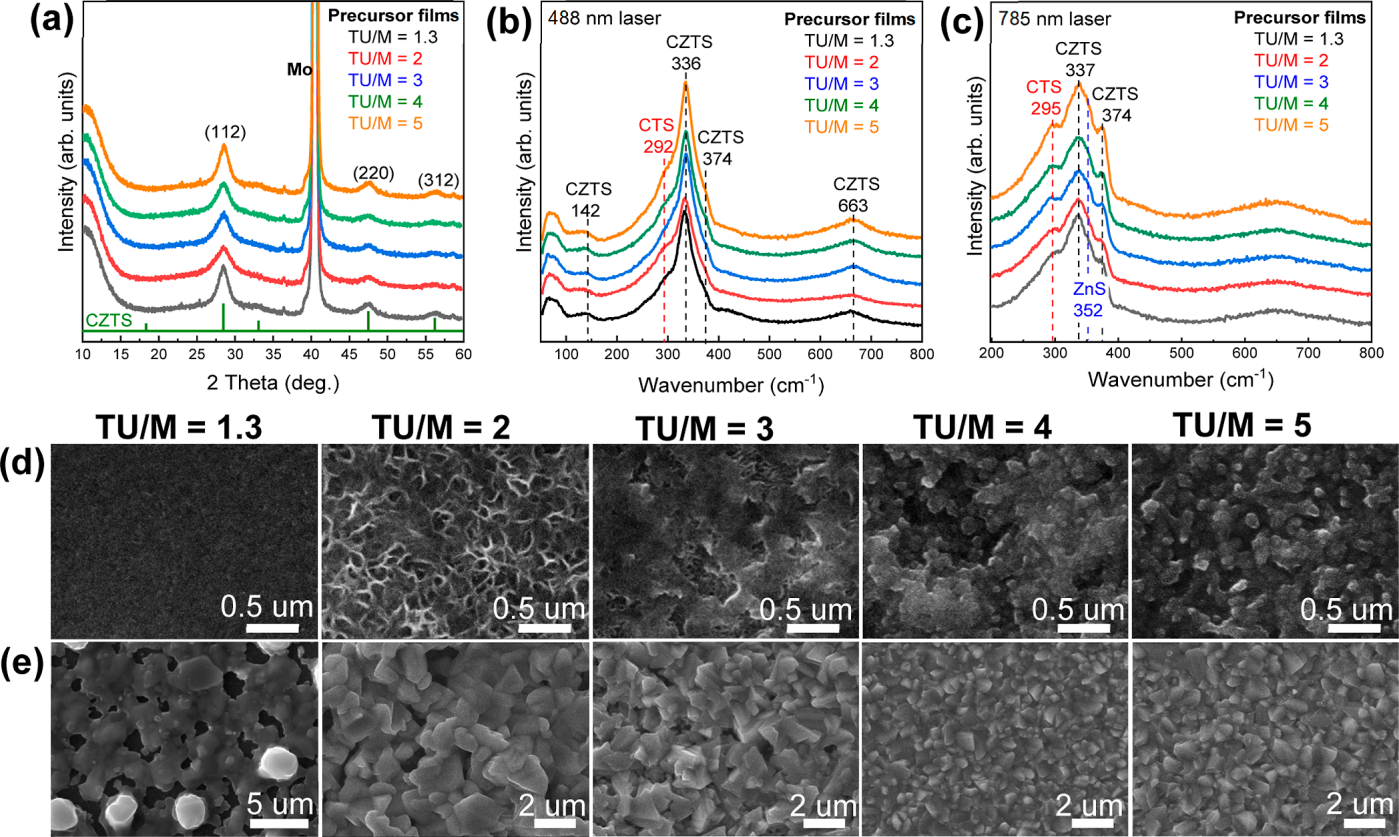
Structure and morphology of thin-films prior and after reactive annealing: (a) X-ray diffractograms and Raman spectra with excitation at 488 (b) and 785 (c) nm of dry precursor films in air at 350 °C with different TU/M ratios. SEM plane view images of thin films prior to (d) and after (e) reactive annealing in the presence of Se at 560 °C.

Figure 2a–c shows the XRD and Raman features of films spin-coated from the precursor solutions with various TU/M ratio and preannealed on a hot plate at 350 °C. The peaks at 28.5° (112), 47.5° (220), and 56.2° (312) can be linked to a range of binary, ternary, and quaternary chalcogenide structures including kesterite, which are observed regardless of the TU/M ratio. Raman spectra recorded with blue (488 nm) and red (785 nm) excitation lasers owing to the increase probability of detection under near resonance conditions, revealed features associated not only with the kesterite lattice but also Cu_2_SnS_3_ and ZnS phases.^29^ Although no distinctive differences are observed in the XRD and Raman analyses of the films, scanning electron micrographs (SEM) images in Figure 2d show that the TU/M ratio has a remarkable effect on the morphology. At TU/M = 1.3, very smooth films with a few nanoscale pores are observed, which are possibly linked to solvent evaporation and byproduct decomposition. Increasing the TU concentration to TU/M = 2 produced a rough mesh-like morphology, while TU/M ≥ 3 resulted in dense films, composed of sintered nanocrystalline grains.

We propose that TU excess in the dry precursor film readily reacts with uncoordinated Sn species to generate SnS_2_, which facilitates the formation of CZTS at the preannealing step, while suppressing SnS_*x*_ loss. Energy-dispersive X-ray (EDX) measurements provided qualitative evidence that the content of C and N in the dry precursor films is largely independent of the TU/M ratio. This was also evident from the intensity of the graphitic carbon peak from the Raman spectra in Figure S2. These surprising results suggest that the benefits brought about by the high TU content do not come at the expense of substantial carbon contamination. Figures 2e and S3 show morphology and cross sections of the CTZSSe films selenized at 560 °C for 20 min. At TU/M = 1.3, SEM results revealed porous films with some large vertically protruding grains identified as Cu_2_S rich phases secondary phases (Figure S4). Both the porosity and secondary phases gradually decrease toward higher TU/M ratio, while no significant changes in Cu/(Zn + Sn) were observed from EDX as summarized in Table S1. At TU/M ≥ 4 reactive selenization under the same conditions generate compact films with average grain size of 1 μm. However, as the TU/M increased, the dense morphology led to a limited rate of Se diffusion, controlled grain growth. Interestingly, SEM images in Figure S5 shows that films obtained with TU/M = 6 are characterized by limited grain growth and nonhomogeneous nucleation, revealing that large TU excess generates very dense dry precursor films which hinders the diffusion of Se during the reactive annealing step. Our observations strongly suggest that the morphology of the dry-precursor films, which is influenced by complexation and self-assembly of molecular species in solution, is key to control the rate of Se diffusion and atomic intermixing during annealing.

### 2.2 Film Optoelectronic Properties and PV Device Performance

**Figure 3:**
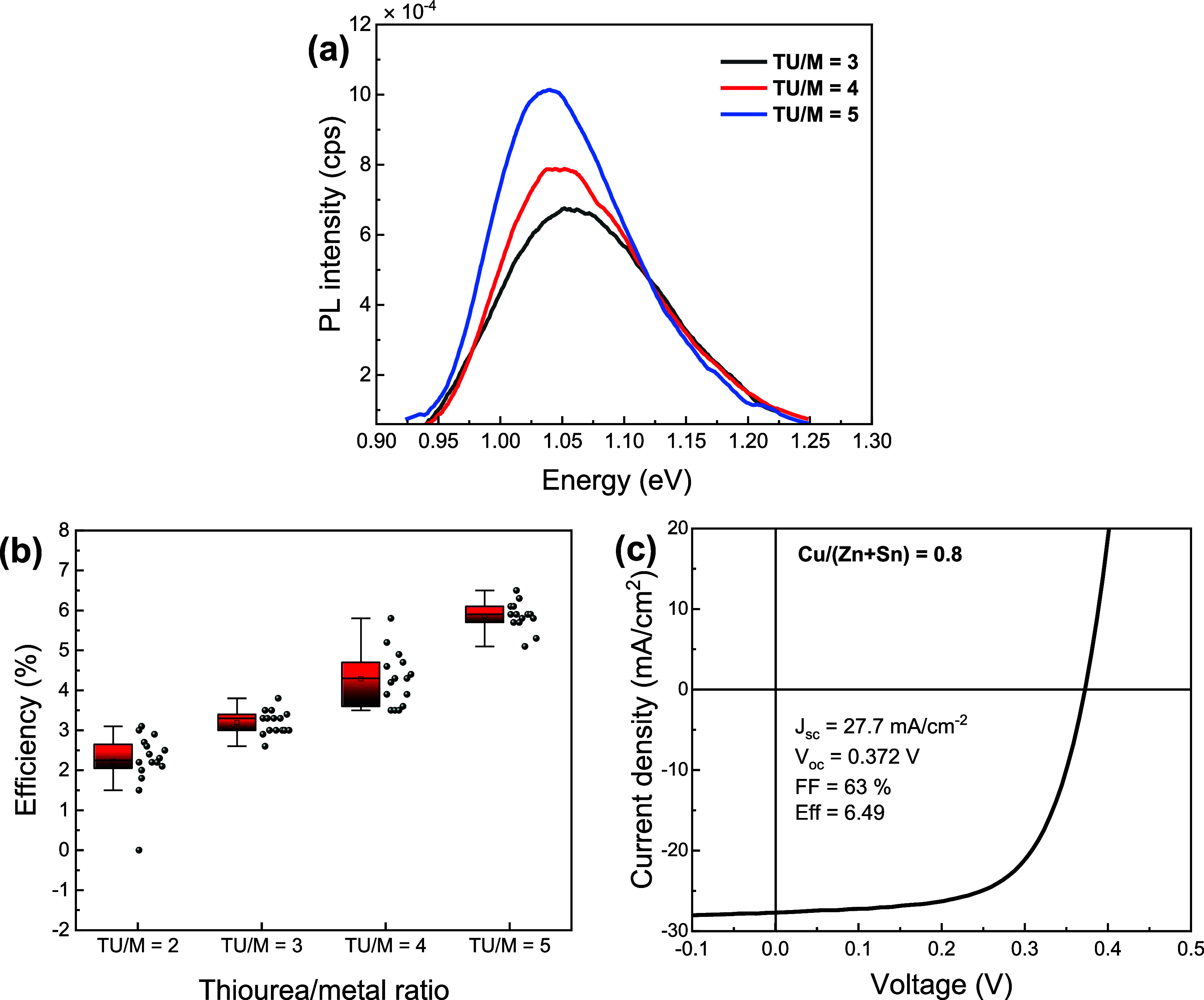
Properties of CZTSSe thin-film devices prepared with various TU/M ratio: (a) Photoluminescence spectra of CZTSSe thin films prepared with TU/M ratios of 3, 4, and 5. (b) PCE box plots of the CZTSSe films with different TU/M ratios. (c) *J*–*V* plot of the best performing cell obtained with a TU/M ratio of 5 and Cu/(Zn + Sn) ratio of 0.8.

Figure 3a shows the room-temperature photoluminescence (PL) responses of the films obtained with various TU/M ratios under 638.3 nm pulsed excitation. The steady-state PL intensity monotonically increases, becoming more symmetric with increasing TU concentration, suggesting a reduction in nonradiative recombination and improved optoelectronic properties. There is an unexpected shift of the PL maximum toward lower energies with increasing TU content, although the values are consistent with the band gap of highly selenized CZTSSe. The band gaps obtained from the inflection point of external quantum efficiency (EQE) plots, shown in Figure S5 and Table S2, were also consistent with the PL values for high TU concentrations (TU/M ≥ 4) but deviated slightly for TU/M = 3 owing to band tails. No PL peaks were obtained for the films with TU/M = 1.3 and 2, indicating high defect density and accelerated carrier decay in the films. The PL characteristics of the film strongly indicate a significant improvement in optoelectronic properties with increasing TU content in the precursor solution. Figure 3b depicts the PCE box plots of devices with substrate architecture: soda-lime-glass/Mo/CZTSSe (0.9 μm)/CdS (50 nm)/*i*-ZnO (50 nm)/AZO (500 nm)/Ag (500 nm), without antireflection coating. Box plots of the photovoltaic parameters are depicted in Figure S6. The mean PCE value clearly increases from 2.2 to 6% with increasing the TU/M ratio from 2 to 5. Figure 3c illustrates the *J*–*V* curves of the best performing devices obtained for a TU/M ratio of 5, while Table 1 summarizes the key corresponding PV parameters of the best cells as a function of TU/M ratio. It is clear from these data that the main improvements are related to *V*_OC_ and FF. This can also be seen in the box plots of the individual photovoltaic parameters in Figure S7. The ensemble of experimental data clearly shows a link between the properties of precursor solutions through the morphology of the dry precursor films and their optoelectronic properties and device performance.

**Table 1:** PV Parameters of Best CZTSSe Thin-Film Devices as a Function of the TU/M Ratio in the Precursor Ink

TU/M	*J*_sc_ (mA cm^–2^)	Voc (V)	FF (%)	eff.(%)
2	27.6	0.25	44	3.08
3	28.2	0.304	44	3.81
4	28.1	0.347	59	5.77
5	27.7	0.372	63	6.49

**Figure 4:**
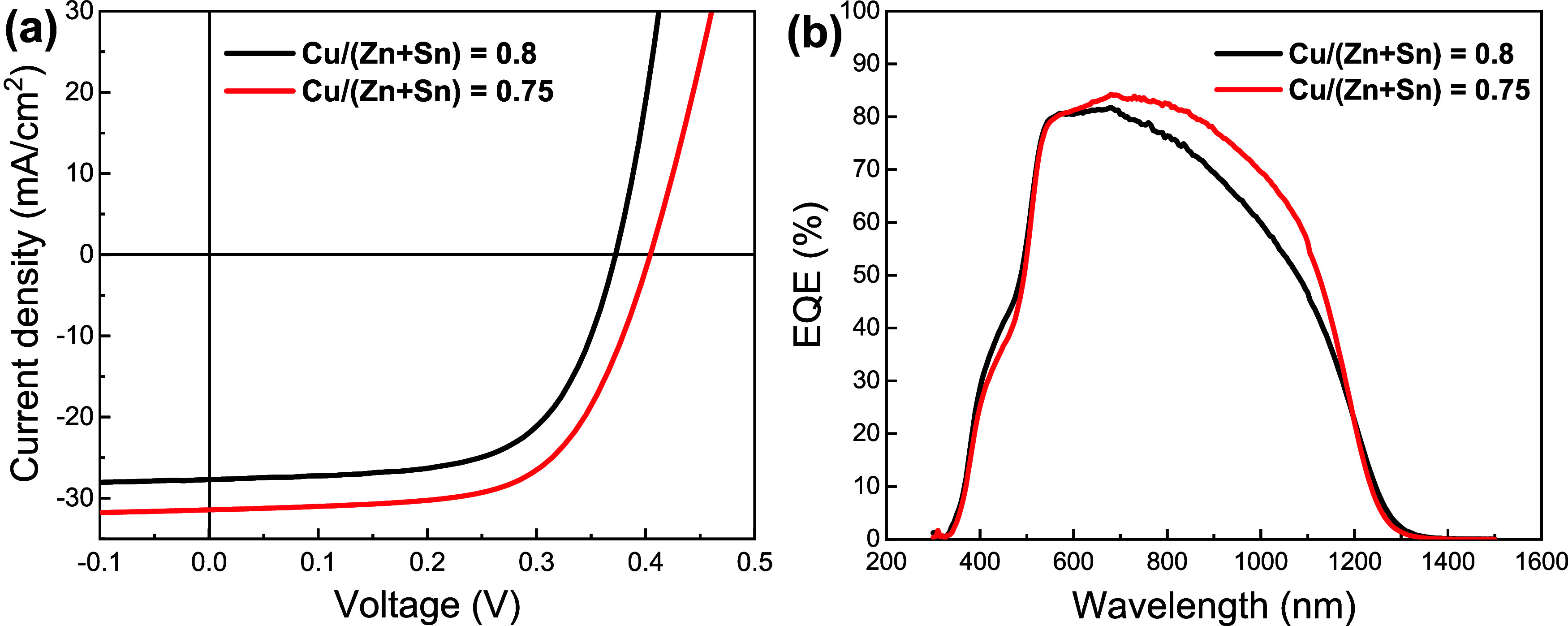
Effect of Cu/Zn + Sn ratio on PV performance of films prepared with TU/M ratio of 5: Characteristic *J*–*V* plot (a) and EQE spectra (b) of the best CZTSSe devices prepared with Cu/Zn + Sn = 0.80 and 0.75.

Figure 4a and Table 2 further show that tuning the Cu content can lead to further enhancement in device performance. The decrease in Cu/(Zn + Sn) ratio from 0.80 to 0.75, while keeping the TU/M at 5, leads to a 13% gain in *J*_SC_ and 8% gain in *V*_OC_, while the FF effectively remains constant, yielding a PCE value close to 8%. The EQE spectra in Figure 4b show an increase in the carrier collection at long wavelengths, which is consistent with lower recombination losses. Interestingly, the Urbach energy extracted from the EQE spectra is higher in the devices with Cu/Zn + Sn ratio 0.75, indicating an increase in cation disorder (Figure S8),^30^ while the Voc deficit decrease by 21 mV. These experiments illustrate the pivotal role of the Cu and TU chemistry in the solution processing of CZTSSe film, from complexation at the solution based molecular precursor to recombination sites at the absorber layer.

**Table 2:** PV Parameters of Devices Fabricated with Different TU/M = 5 and Cu/(Zn + Sn) Ratios of 0.75 and 0.80

Cu/Zn + Sn	*J*_sc_(mAcm^–2^)	*V*_oc_ (V)	FF(%)	eff(%)	*E*_g_ (eV)	E_g_/*q*-*V*_OC_	Eu (meV)
0.80	27.7	0.372	63.0	6.49	1.03	0.658	41.7
0.75	31.4	0.403	62.7	7.95	1.04	0.637	45.2

## 3 Conclusions

We have established for the first time a link between complexation of metal cations and TU in precursor solutions with optoelectronic properties and the device performance of CZTSSe films. FTIR and dynamic light scattering measurements showed that TU strongly interacts with Cu and, to a lesser extent, with Zn forming nanoscale complexes, which self-assemble into colloidal structures in solution with relatively narrow size distribution. The TU/M ratio in the precursor formulation regulates the size of these structures in solution, which, in turn, determines the dynamics of densification and crystallization during the precursor drying step and reactive annealing. Films produced with TU/M = 6 exhibit limited grain growth (Figure S5), while TU/M = 5 generated compact micron size grains with higher PL yield than TU/M values below 3. Increasing the TU/M ratio from 2 to 5 leads to a systematic increase in best PCE values from 3.1 to 6.5%, while decreasing the Cu/(Zn + Sn) ratio from 0.80 to 0.75 led to a further increase of PCE close to 8%. These observations are key in the context of solution processing inorganic thin-film devices, considering the widespread use of TU as a chalcogen precursor in solution processed kesterite and chalcopyrite thin-films. Indeed, the link between complexation, self-assembly, and thin-film microstructure demonstrates that ink formulation in solution processing of compound semiconductors plays a role much more important than just mixing the required elements. The introduction of dopants and other elements, such as Ag and Ge, commonly used for high PCE devices,^31^ can potentially be approached through this methodology given their strong chemical affinity to chalcogen precursors.

## 4 Experimental Details

### 4.1 CZTSSe Precursor and Thin-Film Growth

CZTSSe precursor solutions were prepared based on the approach reported by Tiwari et al.,^25^ which involves the sequential dissolution of CuCl_2_·2H_2_O (≥99%, Sigma-Aldrich), SnCl_2_ (98%, Sigma-Aldrich), ZnCl_2_ (≥98%, Sigma-Aldrich), and TU (99+% Sigma-Aldrich) in a 50:50 vol % DMF/IPA binary solvent in the ratios Cu/Zn + Sn = 0.8 and 0.75, Zn/Sn = 1.2 and TU/metal = 1.3, 2, 3, 4, and 5. Each salt was sonicated for 15–20 min to ensure complete dissolution before the next. Before each dissolution step, the salt was added to the solvent in a glovebox and sealed tight, followed by ultrasonication in air. Prior to spin-coating the molecular precursor, molybdenum-coated soda lime glass substrates (AimCore) were cleaned by sequential sonication in acetone, isopropanol, and water and then UV–ozone treated for 20 min. Spin-coating was carried out at 3000 rpm for 60 s, followed by preannealing (drying) step on a hot plate at 350 °C for 2 min. The process was repeated 13 times to achieve a film thickness of about 0.9 μm. The films were subsequently loaded into a graphite box together with 0.5 g of selenium powder, heated in a rapid thermal annealing furnace at a ramp rate of 1.8 °C/s until 560 °C, and held at this temperature for 20 min under a constant flow of ultrapure argon at 28 sccm and a maintained pressure of 1 atm.

### 4.2 Device Fabrication

Complete devices were fabricated in the substrate configuration, beginning with 50–60 nm thick CdS deposited on top of the CZTSSe absorber via chemical bath deposition, a window layer consisting of 50 nm of *i*-ZnO and 500 nm of AZO deposited by RF-sputtering, and 500 nm of Ag top electrode deposited by thermal evaporation. No antireflection coating was employed.

### 4.3 Characterization Tools

Raman spectra were collected from at least four different locations on the thin films and on three different drops of solutions using the PerkinElmer RamanFlex 400 with an excitation wavelength of 785 nm and Renishaw inVia at 488 nm. Two different excitation wavelengths were employed owing to the increased probability of detection under near resonance conditions, where the wavelength of the excitation source approaches the band gap of the compound of interest. Prior to the measurements, calibration was performed with a polystyrene reference. Metal–organic interactions were probed by FTIR spectrophotometer with the PerkinElmer FTIR Spectrometer Spectrum Two, equipped with a diamond crystal in attenuated total reflection geometry at a 4 cm^–1^ resolution. X-ray diffractograms were recorded using a Bruker D8 Advance instrument equipped with a Cu Kα source (λ = 1.5418 Å) and a PSD LynxEye detector. SEM was performed with a Jeol IT300SEM at 15 kV accelerating voltage and 20 mA probe current. Photoluminescence measurements were measured at room temperature using a 40 MHz pulsed 638.3 nm laser with an Oriel cornerstone 130 1/8m monochromator. Current–voltage (*J*–*V*) responses were measured on 0.25 cm^2^ total cell area under an AM 1.5 G spectrum using a solar simulator (WAVELABS Sinus-70 light) and source meter (Keithley 2400 standard series). EQE measurements were performed on a PVE300 system (Bentham TMc300), with a monochromator, a dual halogen and single xenon as light sources, and a transformer (x500 474 type preamp).

## Data Availability

Data are available at the University of Bristol data repository, data.bris, at https://doi.org/10.5523/bris.1vzv31fd26bvv2w8grbbmonrlt.

## Supporting Information Available

FTIR spectra of TU/CuCl2 and TU/ZnCl2 solutions with 1.3 TU/M ratio; Raman spectra of CZTSSe thin films prepared with various TU/M ratios in the 1100–1800 cm^–1^ range; SEM cross sections of CZTSSe absorbers with TU/M = 1.3 to 5; SEM—EDX maps of selenized CZTSSe absorber with TU/M = 1.3; SEM images of CZTSSe films with TU/M ratio = 6; band gap analysis based on photoluminescence and EQE spectra of CZTSSe thin-film devices as a function of TU/M ratio in the precursor solution; statistically variations of photovoltaic parameters from CZTSSe cells prepared with various TU/M ratios in the precursor solution; estimation of Urbach energy tails from the EQE spectra of devices with Cu/Zn + Sn = 0.8 and 0.75; atomic composition of CZTS precursor films with different TU/M ratios; and band gap (E_g_) estimations based on PL and EQE of the CZTSSe absorbers obtained with various TU/M ratios in the precursor solution (PDF)
