# Supplementary material for: Correlating Molecular Precursor Interactions with Device Performance in Solution-Processed Cu2ZnSn(S,Se)4 Thin-Film Solar Cells
Source: ACS Appl Mater Interfaces. 2024 Jun 27;16(27):35315–22. doi: 10.1021/acsami.4c05321 (PMC11247423; doi:10.1021/acsami.4c05321)
Supplement: Supplementary file 1 — am4c05321_si_001.pdf [file am4c05321_si_001.pdf]

Correlating molecular precursor interactions with device performance in solution-processed $CuZnSn(S,Se)_4$ thin-film solar cells

Raphael Agbenyeke[1], Alice Sheppard[1], Jacques Keynon[2], Nada Benhaddou[2], Nicole Fleck[3], Valentina Corsetti,[1] Mohammed A. Alkhalifah[1,4], Devendra Tiwari[3], Jake W. Bowers[2], and David J. Fermin[1]*

[1]School of Chemistry, University of Bristol, Bristol BS8 1TS, United Kingdom.

[2]Centre for Renewable Energy Systems Technology (CREST), Wolfson School of Mechanical, Electrical and Manufacturing Engineering, Loughborough, LE11 3TU, United Kingdom.

[3]Department of Mathematics, Physics and Electrical Engineering, Northumbria University, Ellison Building, Newcastle Upon Tyne, NE1 8ST, United Kingdom.

[4] Department of Chemistry, College of Science, King Faisal University, Al-Ahsa, 31982, Saudi Arabia.

*Corresponding Author

Prof. David J. Fermin – David.Fermin@bristol.ac.uk

Content

Figure S1. Figure S1. FTIR spectra of $TU/CuCl_2$ and $TU/ZnCl_2$ solutions with 1.3 TU/M ratio.

Figure S2. Raman spectra of CZTSSe thin-films prepared with various TU/M ratios in the 1100 to 1800 cm^{-1}.

Figure S3. SEM – EDX maps of selenized CZTSSe absorber with TU/M = 1.3.

Figure S4. SEM cross-sections of CZTSSe absorbers with TU/M = 1.3 to 5.

Figure S5. SEM images of CZTSSe films with TU/M ratio = 6.

Figure S6. Band gap analysis based on photoluminescence and external quantum efficiency (EQE) spectra of CZTSSe thin-film devices as a function of TU/M ratio in the precursor solution.

Figure S7. Statistically variations of photovoltaic parameters from CZTSSe cells prepared with various TU/M ratio in the precursor solution.

Figure S8. Estimation of Urbach energy tails from the EQE spectra of devices with Cu/Zn+Sn = 0.8 and 0.75.

Table S1. Atomic composition of CZTS precursor films with different TU/M ratios

Table S2. Band gap (E_g) estimations based on PL and EQE of the CZTSSe absorbers obtained with various TU/M ratios in the precursor solution.

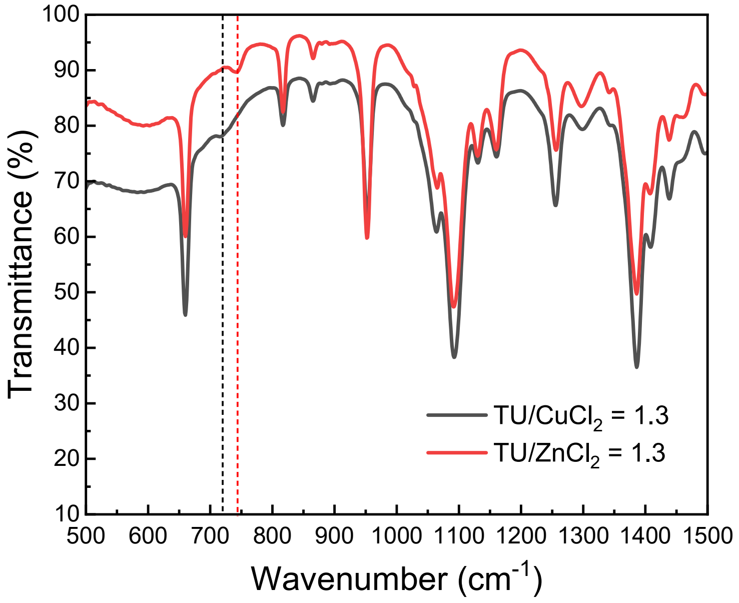

Figure S1. FTIR spectra of TU/$CuCl_2$ and TU/$ZnCl_2$ solutions with 1.3 TU/M ratio. A chemical shift in the TU C=S peak is observed in the range of 700 to 750 cm^{-1} in the presence of Cu, while no shift is observed in the case of Zn.

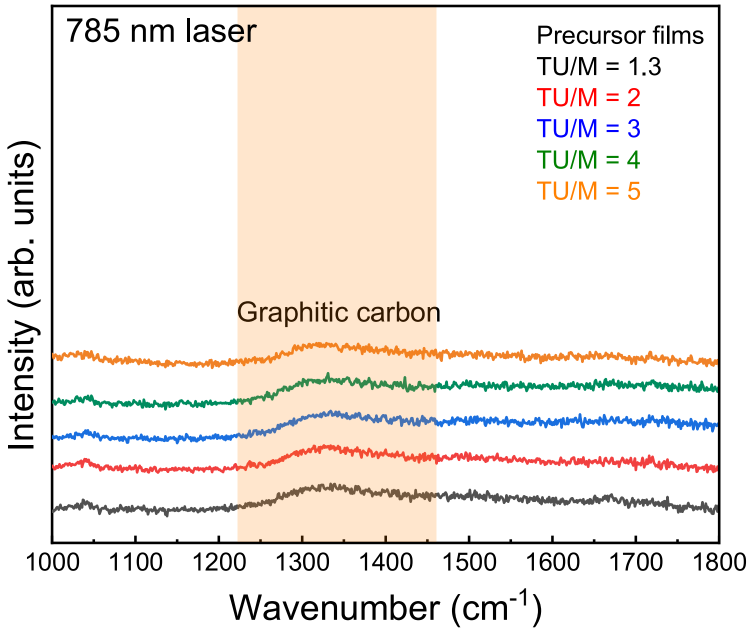

Figure S2. Raman spectra of annealed CZTSSe thin-films prepared with various TU/M ratios in the 1100 to 1800 cm^{-1}. All spectra show a very weak graphitic carbon signal, suggesting no significant carbon accumulation with increasing TU in the precursor solution.

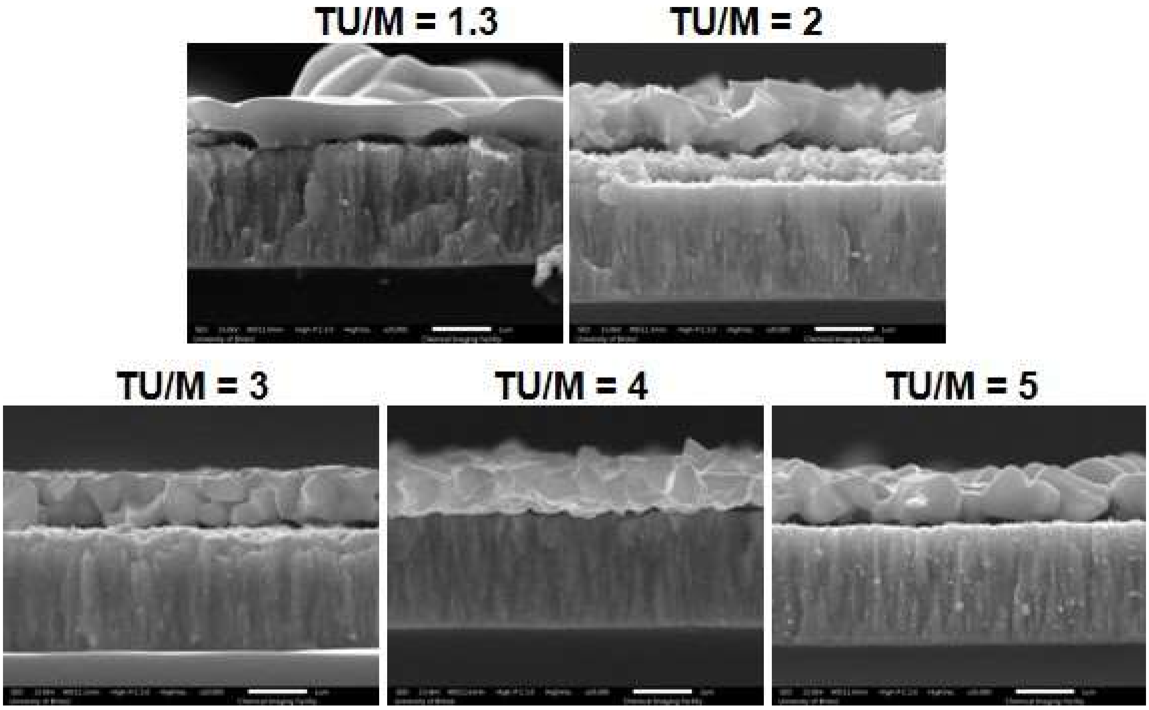

Figure S3. SEM cross-sections of CZTSSe absorbers with TU/M = 1.3 to 5.

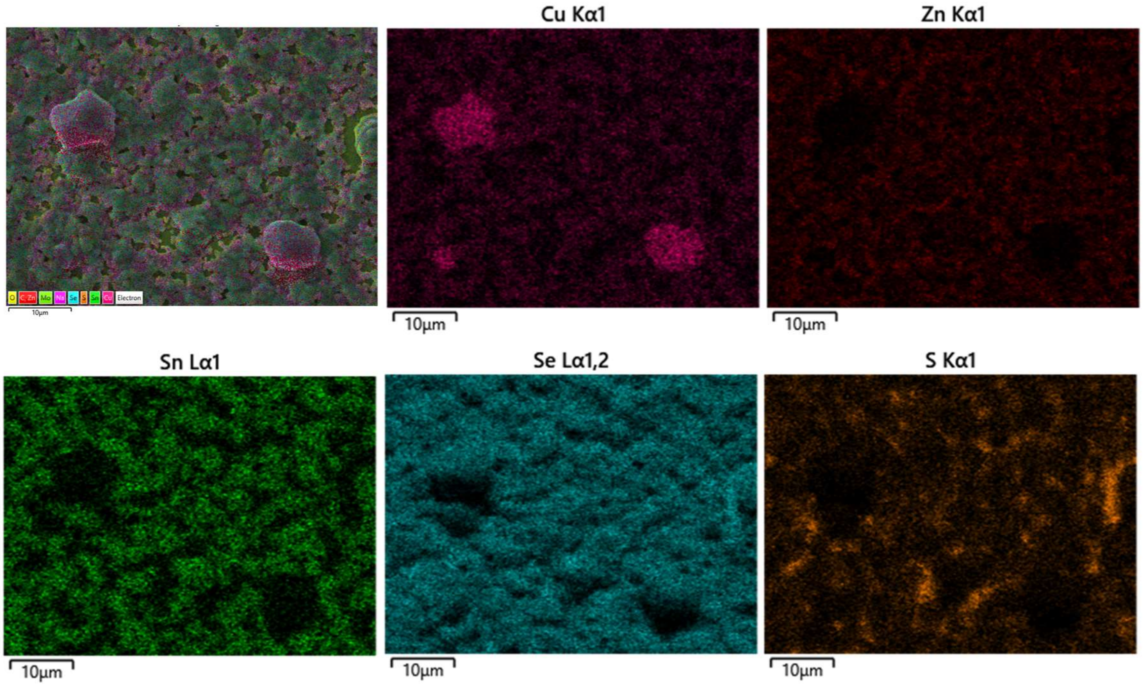

Figure S4. SEM-EDX maps of selenized CZTSSe absorber with TU/M = 1.3.

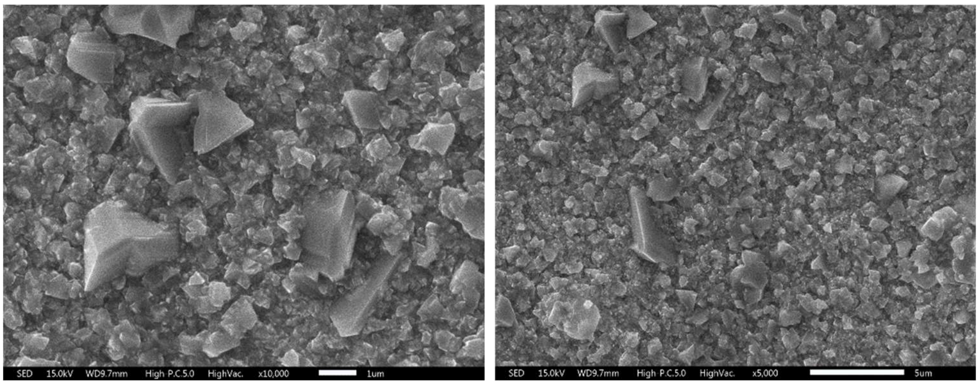

Figure S5. SEM images of CZTSSe films with TU/M ratio = 6. The limited grain growth and non-homogenous nucleation can be attributed to high packing density of the precursor films which hinders the diffusion of Se during reactive selenization.

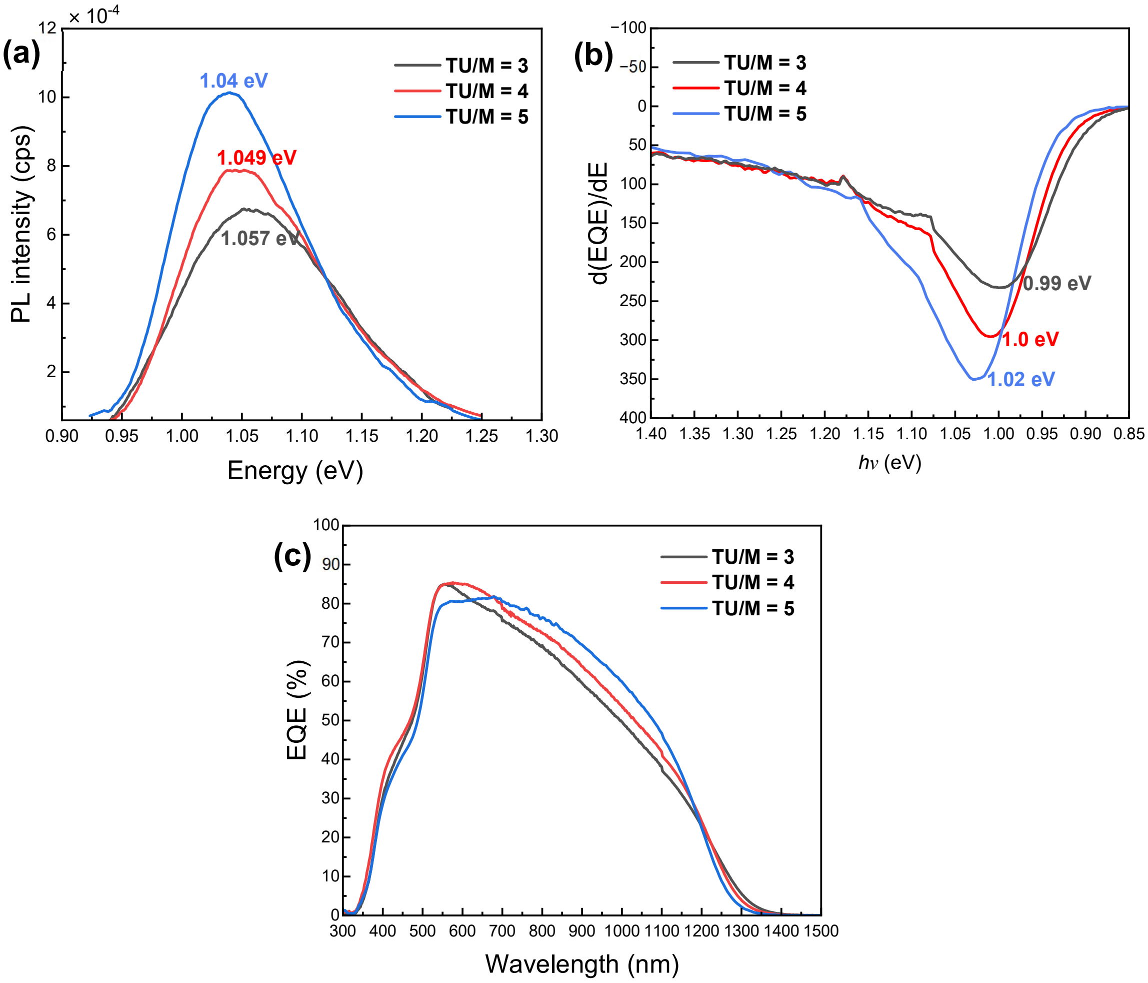

Figure S6. Band gap analysis based on photoluminescence (PL) and external quantum efficiency (EQE) spectra of CZTSSe thin-film devices as a function of TU/M ratio in the precursor solution. (a) PL spectra (b) derivative of EQE with wavelength and (c) EQE spectra of CZTSSe absorbers.

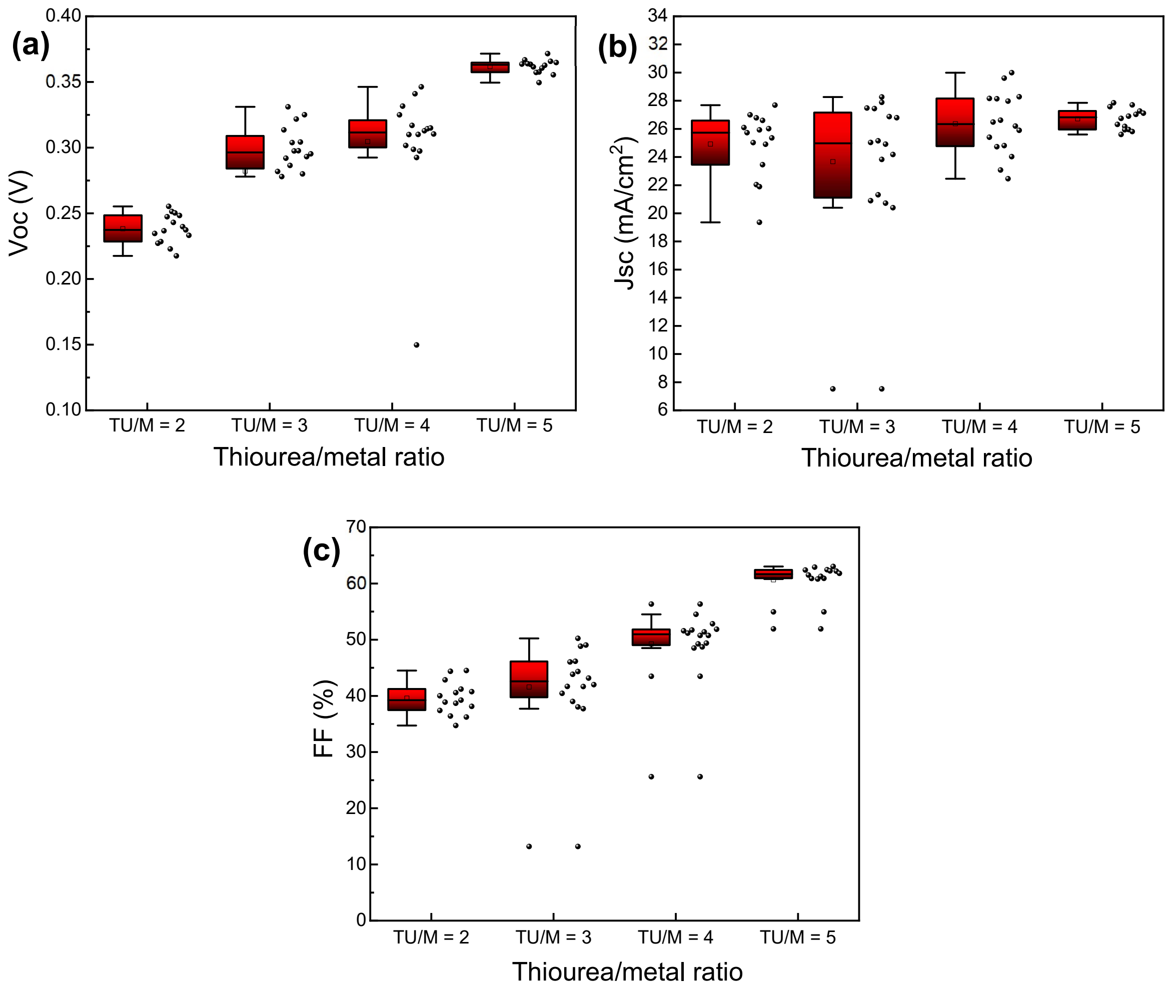

Figure S7. Statistically variations of photovoltaic parameters from CZTSSe cells prepared with various TU/M ratio in the precursor solution. (a) open circuit voltage (V_{OC}), (b) Short-circuit current (J_{SC}), (c) fill factor (FF). V_{OC} and FF significantly improve with increasing TU/M ratio while J_{SC} shows a very weak dependence.

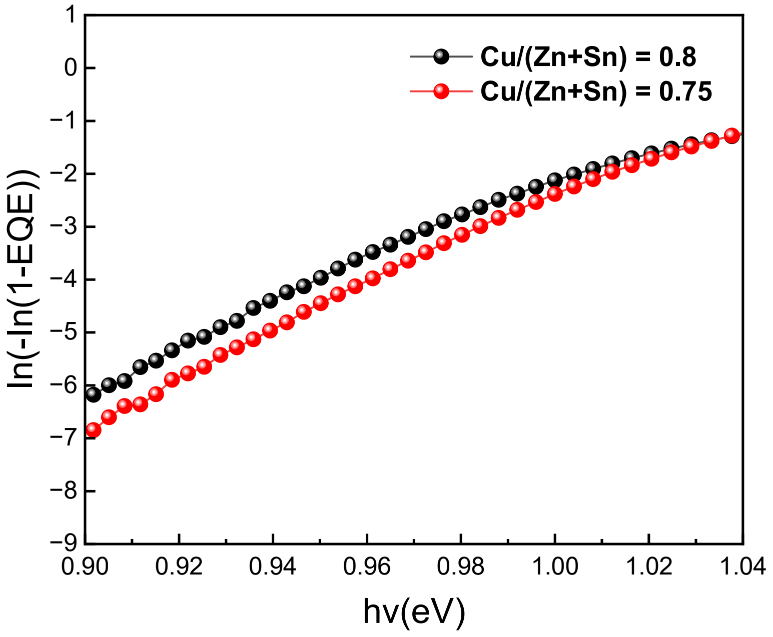

Figure S8. Estimation of Urbach energy tails from the EQE spectra of devices with Cu/Zn+Sn = 0.8 and 0.75.

Table S1. Atomic composition of CZTS precursor films with different TU/M ratios

TU/M	Atomic percent			Cu/(Zn+Sn)
	Cu	Zn	Sn	
1.3	9.9	6.5	4.1	0.93
2	7.9	5.3	4.8	0.78
3	8.0	5.4	4.8	0.78
4	7.8	5.1	5.0	0.77
5	7.9	5.3	4.8	0.78

Table S2. Band gap (E_g) estimations based on PL and EQE of the CZTSSe absorbers obtained with various TU/M ratios in the precursor solution.

TU/M	E_g PL / eV	E_g EQE / eV	E_g (PL-EQE) / eV
3	1.06	0.99	0.07
4	1.05	1.00	0.05
5	1.04	1.02	0.02
